# Social status and prenatal testosterone exposure assessed via second-to-fourth digit ratio affect 6–9-year-old children’s prosocial choices

**DOI:** 10.1038/s41598-018-27468-0

**Published:** 2018-06-15

**Authors:** Lisa Horn, Niklas A. Hungerländer, Sonja Windhager, Thomas Bugnyar, Jorg J. M. Massen

**Affiliations:** 1 Department of Cognitive Biology, University of Vienna, Vienna, Austria; 2 Department of Anthropology, University of Vienna, Vienna, Austria; 3 Department of Theoretical Biology, University of Vienna, Vienna, Austria

## Abstract

Prosocial behaviour (i.e., voluntary behaviour intended to benefit another) seems to be fully developed in children by the age of 6 years. However, questions about which factors modify prosocial behaviour at that age remain understudied. Here we used a resource allocation paradigm to test prosocial behaviour in 6–9-year-old school children. They could decide between a “selfish” (i.e., one sticker for themselves) and a “prosocial” option (i.e., one sticker for themselves and one for the receiver) and we tested whether friendship, social status and prenatal androgen exposure (approximated by the 2nd to 4th digit ratio; 2D:4D) influenced children’s prosocial choices. We found that children behaved prosocially, and that their prosocial tendencies were negatively correlated with prenatal androgen exposure; i.e., children with high 2D:4D ratios (reflecting low prenatal androgen exposure) acted more prosocially than children with low 2D:4D ratios. Further, their social status in the classroom influenced their choices: children with fewer interaction partners chose the “prosocial” option more often than more ‘popular’ children. However, they did so irrespectively of whether they were paired with a recipient or not. Our results highlight the importance of considering social, as well as physiological factors when investigating prosocial behaviour in children.

## Introduction

To help, cooperate, and share with others are central aspects of human societies, and they range from large-scale cooperation to small acts of charity. How this prosocial behaviour (i.e. “voluntary behavior intended to benefit another”^1^, p. 646) develops during ontogeny has recently become a hot topic of developmental psychology – so much so that more than 20% of studies on prosocial behaviour in children have been published in the last 6 years^2^. These studies suggest that prosocial behaviour emerges early in human ontogeny, with some researchers arguing that it forms a biological predisposition^3,4^. Already by the age of 14 months infants help in simple tasks, such as handing an experimenter an out-of-reach object^5^. At this age, prosocial behaviour is indiscriminate of the other’s identity and limited to simple helping tasks. By the age of three, children start behaving more selectively by punishing or withdrawing cooperation from uncooperative social partners^6^. Sharing resources with others develops even later in childhood, as children’s prosocial tendencies increase with experience and they are more strongly influenced by social norms^7,8^.

One way to experimentally assess children’s propensity to share with others are resource allocation paradigms inspired by behavioural economics: in these “games” a participant (i.e. the donor) is presented with two options that have different payoffs for him- or herself and a partner (i.e. the recipient). In a typical *prosocial game* (cf. prosocial choice task)^9^ the donors can choose between one option, where they receive a reward and simultaneously deliver a reward to the recipient (1/1 option), and a second option, where only they themselves receive a reward (1/0 option). In this game there are no costs for the donor, as he or she will always receive one reward item, regardless of their choice. In comparison, in a *costly sharing game*, the donors are required to choose between the altruistic 1/1 option and a 2/0 option, which would deliver two reward items to them. Consequently, in this game, the donor has to forego maximizing his or her own payoff in order to benefit the recipient.

Brownell and colleagues^10^ found that in a cost-free *prosocial game*, 2-year-old children only showed a preference for resource distributions that benefitted an adult recipient, when the recipient verbally expressed a desire for the reward. House and colleagues^11^ tested 3–8-year-old children with peers (i.e., familiar children from their classroom) as recipients in both the *prosocial* and the *costly sharing game*. They found that the children had a tendency to share in the *prosocial game*, but only when discarding those trials in which the donors laughed while making their choice. In such trials the donors predominantly chose the self-serving option. Also, they chose the prosocial option more often in the test condition, where the donor could actually benefit a recipient, compared to a control condition, where no recipient was present. However, this effect was mainly driven by the 7–8-year-olds. This suggests that only from that age on children make conscious choices about sharing. Recipient requests for the reward occurred in only a few cases in this study and are therefore not likely to have influenced the donors’ prosocial giving^11^. These results parallel earlier findings conducted in an anonymous setting (i.e. recipient’s identity only represented by a classroom photo)^12^: while at age 3–4 many children in this study acted selfishly, by 7–8 years the children shared equally with the recipient irrespective of costs for themselves, presumably due to a preference for equal outcomes.

While prosocial sharing had long been regarded a hallmark of human behaviour, recent studies found prosocial tendencies in various non-human primate species (e.g.^13–16^, for a review see)^17^, dogs^18^ and birds^19^. Investigating in which extant species prosociality occurs (and in which not) and how non-human prosocial tendencies compare to human ones allows researchers to hypothesize about the evolutionary foundations of human prosociality (e.g. a potential positive effect of cooperative child-rearing on prosocial tendencies in early human societies, cf.^20^). However, a prerequisite is testing all species with comparable methods. Recently, two studies^21,22^ tested children in resource allocation paradigms comparable to those used in non-human primates^15,23^. They used an apparatus that had physical properties that allowed the donor to choose only one option per trial, thereby avoiding elaborate experimenter instructions. In one study^21^, the 1.5–5-year-old children only behaved prosocially when they had to choose between an option delivering a reward to the recipient (0/1 option) compared to the alternative where there was no reward for either the donor or the recipient (0/0 option). However, in a standard *prosocial game* (1/1 vs. 1/0) the same children did not take the recipient’s payoff into account. In the other study^22^, where the donor could decide between delivering a high-quality reward or a low-quality reward to the recipient, 7-year-old children chose to deliver the high-quality reward to the other child only when they themselves also received the high-quality reward. In this respect, their behaviour was comparable with that of adult chimpanzees tested in the same study. In contrast, 5-year-old children did not behave prosocially at all in this study^22^. Both studies used a side-by-side set-up, where donor and recipient were positioned next to each other and the trays with the rewards were placed in front of them. It is possible that this set-up introduced attentional problems, because the children were so focused on their own payoff that they disregarded the reward distribution on the recipient’s side^21^.

Nevertheless, it is also possible that children’s prosocial behaviour is not indiscriminate and depends on the characteristics of the donor, the recipient, or both^24^. One of the potential features to influence prosocial tendencies is the quality of the relationship between donor and recipient. Adults believe that prosocial behaviour is an important element of friendship^25^ and preschool children already expect more prosocial behaviour between friends than between non-friends^26,27^. Moreover, elementary school children are more sociable and cooperative with friends than with peers with whom they have less affectionate ties^28^, and similar patterns have been found among non-human animals^29–31^. Moore^32^ found that friendship influenced 4.5–6-year-old children’s choices in a resource allocation experiment. The children had to name a friend with whom they liked to play and a non-friend with whom they did not prefer to play at all. They shared more often with the friend than with the non-friend, with allocations to an unfamiliar child taking an intermediate level. However, these children were not tested in real interactions with their peers, and instead, drawings were used to represent the identity of the recipient. In direct peer interactions, some studies did not find a difference in prosocial donating between friends and acquaintances^33^, or even a reduced preference to benefit friends, if the interaction was framed in a competitive context^34,35^. Therefore, it is important to investigate resource allocations in the presence of a familiar peer. Interestingly, using such a choice paradigm with direct interactions, long-tailed macaques do not necessarily choose to benefit their friends, and their choices seem more influenced by status effects (i.e., dominance hierarchy)^13,14^.

Among children, social status can be defined as a summary measure of the degree to which a group member is liked or disliked by peers as a whole^36^. This popularity can be assessed by the number of peer nominations as best friend or by the number of interaction partners during free play^37,38^. Moreover, the higher an individual’s social status in the group, the more the other children pay attention to this individual^37–39^. A further key aspect of social status is social dominance, which has been described as the ability to acquire valuable resources, regardless of whether they are obtained by coercive behaviour or prosocial, cooperative interactions^40^. It is not clear if prosocial behaviour is used more by individuals with a high or with a low social status in the group. High-status individuals could use prosocial behaviour in order to consolidate their social rank (cf.^13^), and in turn, prosocial behaviour could lead to high status^41^. On the other hand, low-status individuals could use prosocial behaviour in order to avoid sanctions from dominant individuals^40^ (cf.^14^). In line with the second argument, a recent study showed that children with a low social status – determined by a previous resource competition experiment – donated more rewards to an anonymous recipient than children with high social status^42^.

One final aspect that has been underrepresented when investigating prosocial behaviour is a potential physiological determinant. In adult humans it has been shown that prosocial behaviour in economic games is influenced by administration of testosterone, with some studies showing testosterone to decrease prosocial behaviour (e.g.^43^), while others demonstrate increased prosocial tendencies after testosterone administration (e.g.^44^). Additionally, the effects of prenatal androgen exposure seem to independently modulate prosocial behaviour later in life^45^. Prenatal androgen exposure can be approximated non-invasively, by measuring the second-to-fourth digit (2D:4D) ratio^46^. While the early foundations of the 2D:4D approach to prenatal testosterone exposure relied heavily on correlational inference (e.g., sexually dimorphic 2D:4D ratios, cord blood measures), Zheng and Cohn^47^ demonstrated the developmental and molecular pathways of the association between prenatal testosterone exposure and the 2D:4D ratio in a mouse model. Moreover, several studies have found that a direct manipulation of prenatal androgen levels, or a manipulation of the binding potential of prenatal androgens with the receptors of the embryo(s) leads to differences in adult 2D:4D ratios in the predicted directions in both mice and rats^48–50^. However, it has remained controversial whether 2D:4D is mainly determined by prenatal testosterone, or by a balance of prenatal testosterone relative to prenatal estrogen^46^, and effects as well as effect sizes in relation to other investigated traits are currently still debated.

While the ratio increases with age at least until early adulthood, the rank order is relatively stable across the life span^51,52^. Importantly, the 2D:4D ratio of the right hand has been argued to show a stronger association with prenatal androgens than that of the left hand^53^. Individuals with high prenatal androgen exposure have a lower 2D:4D ratio, while individuals with low exposure have a relatively higher 2D:4D ratio. Although women tend to have a larger 2D:4D ratios than men, between-sex overlap and within-sex variability are usually large^54^. The variation in 2D:4D ratios has been closely related to variation in gender-typed appearance and behaviours within each sex (for a review see^55,56^). Males with higher 2D:4D ratios have a less masculine behavioural phenotype^57^ and less physical strength compared to those with lower 2D:4D^58^. Females with lower 2D:4D, in turn, score higher on male-dominated dimensions, such as spatial abilities^59^ and a systemizing personality^60^, than same-sex individuals with a higher 2D:4D ratio. Therefore, one can argue for a general masculinisation effect of prenatal androgen exposure on multiple phenotypic levels, from brain organization to appearance and behaviours.

In 5–7-year-old children, prosocial tendencies scored via a teacher questionnaire were positively correlated with their 2D:4D ratios, suggesting that children with high 2D:4D ratios (indicating lower prenatal androgen exposure) were perceived as more prosocial^61^. Millet and Dewitte^62^ found that adults with lower 2D:4D ratios (indicating higher prenatal androgen exposure) were more likely to give a fair share and less likely to give either more or less than the fair share in a public goods game, where multiple participants could contribute to a shared financial pay-off. In a further study, the authors investigated how much money the participants would donate to an anonymous recipient^63^. Here, participants with lower 2D:4D ratios actually donated more money. However, after exposure to aggression cues, this relationship was inverted and subjects with high 2D:4D ratios acted more prosocially^63^. Notwithstanding the results above, to date there are no experimental studies investigating the connection between prenatal androgen exposure and allocations in a resource allocation experiment in children.

**Figure 1:**
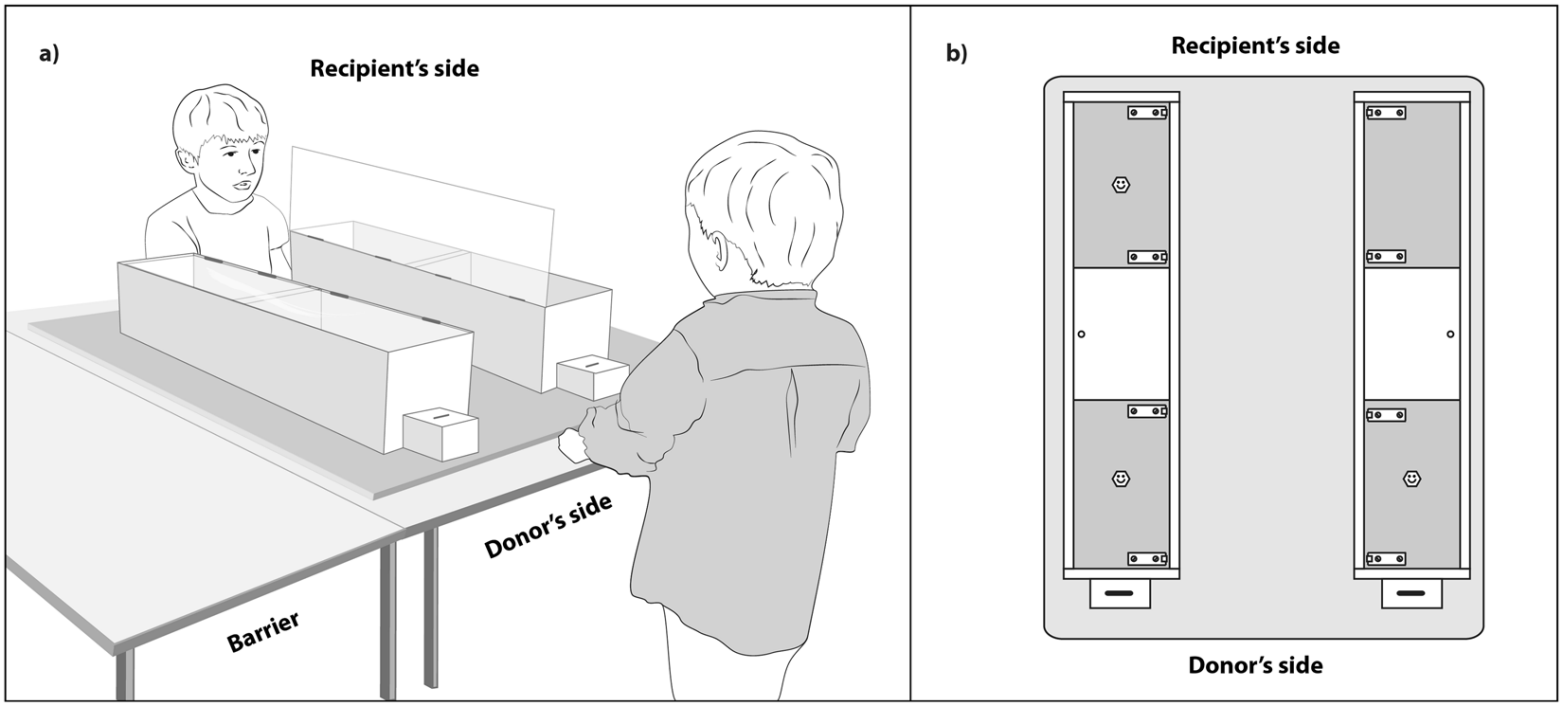
(**a**) Drawing of the experimental set-up of the prosocial test condition. The apparatus is placed on the tables that serve as a barrier between the two sides. The donor child is on the right side. By putting a coin in one of the small coin receptacles on his side, the donor can open the connected larger reward box. The illustration shows the apparatus after the donor chose the right reward box, whose lid is open. Illustration by Nadja Kavcik-Graumann. (**b**) Schematic bird’s eye view of the experimental apparatus. The hexagons represent the reward items (i.e., stickers). The 1/1 option is on the left side and the 1/0 option is on the right side.

The aim of the current study was to investigate 6–9-year-old children’s prosocial tendencies in a resource allocation paradigm that, comparable to animal studies, was constrained by the apparatus’ physical properties and not by rules established by the experimenter. To avoid attentional biases (cf.^21^), we used a set-up where the two children were facing each other instead of a side-by-side set-up (Fig. 1). We used a *prosocial game* to assess the children’s propensity to choose the 1/1 option (i.e., one reward item on their side and one reward item on the recipient’s side) versus the 1/0 option (i.e., one reward item on their side, but no reward item on the recipient’s side) in the prosocial test condition when a recipient was present compared to a non-social control condition when they were tested alone. The experiment started with a warm-up phase, where the donor children could learn how to operate the apparatus and understand the consequences of their choices (cf.^21,22^). Each donor child was then paired with one familiar, same-sex recipient from their classroom and received 10 consecutive trials in both the test and the non-social control (sequence of conditions counterbalanced across children). Further, we tested whether friendship, social status and prenatal androgen exposure (approximated by their 2D:4D ratio) influenced children’s prosocial behaviour in this task. Friendship was assessed by questionnaires given to the children and the classroom teachers. Half of the children were paired with a recipient that they indicated as a friend, the other half was paired with a recipient that they had not indicated as a friend. Social status was assessed live during free play observations, where we scored each child’s average number of interaction partners and number of peers paying attention to the child. Additionally, classroom teachers were asked to score the children’s perceived social dominance. Digit ratios were measured from hand scans made prior to the behavioural experiment. We tested 48 children from 4 different classrooms. Three children failed the warm-up in the resource allocation experiment and were excluded from data analysis. Of the 45 remaining children, seven children were missing data from one other part of the study (see methods section for details). In the results section, we report analyses on the maximum sample size per variable (see also Table 1). We also analysed the reduced sample from only those children with a complete data set and the results are fully equivalent to the maximum sample (see Supplementary Results file).

**Table 1:** Sample size and descriptive statistics (mean, standard deviation, median, minimum, maximum) on all variables, split by sex and for the total sample.

Sex			1/1 choices Prosocial test	1/1 choices Non-social control	Prosocial tendencies	Interaction partners	Other children attending	Dominance rating	2D:4D ratio	Age (mo)
male	N	valid	19	19	19	19	19	19	17	18
		missing	0	0	0	0	0	0	2	1
	mean		7.53	5.79	1.74	1.62	0.87	50.86	0.954	97.22
	sd		2.590	2.551	3.280	1.156	0.642	26.624	0.036	9.950
	median		9.0	6.0	1.0	1.3	0.8	50.0	0.951	97.5
	minimum		3.0	1.0	−3.0	0.3	0.0	15.5	0.896	82.0
	maximum		10.0	10.0	9.0	4.0	2.0	92.3	1.028	117.0
female	N	valid	26	26	26	25	25	26	24	25
		missing	0	0	0	1	1	0	2	1
	mean		7.85	6.15	1.69	1.55	0.90	52.46	0.959	95.04
	sd		1.736	2.767	2.724	0.445	0.289	24.739	0.030	8.919
	median		8.0	6.5	1.0	1.5	1.0	54.1	0.961	95.0
	minimum		4.0	0.0	−3.0	0.8	0.3	6.8	0.909	80.0
	maximum		10.0	10.0	8.0	2.5	1.5	99.8	1.017	112.0
total	N	valid	45	45	45	44	44	45	41	43
		missing	0	0	0	1	1	0	4	2
	mean		7.71	6.00	1.71	1.58	0.89	51.78	0.957	95.95
	sd		2.117	2.654	2.936	0.819	0.468	25.265	0.032	9.312
	median		8.0	6.0	1.0	1.5	0.8	52.8	0.955	95.0
	minimum		3.0	0.0	−3.0	0.3	0.0	6.8	0.896	80.0
	maximum		10.0	10.0	9.0	4.0	2.0	99.8	1.028	117.0

We predicted that 6–9-year-old children would choose the prosocial 1/1 option more when a peer was present as a recipient than when tested alone. Further, we predicted that the children would share more when they were paired with a friend than when they were paired with a non-friend^32^. Regarding the connection between social status and prosocial tendencies, we hypothesized that children with a lower social status would behave more prosocially^42^. If the effects of prenatal testosterone exposure (assessed via 2D:4D) modulate the children’s choices, two competing hypotheses can be phrased in the light of the existing literature. The first line of argument is based on the overall masculinising effect with increasing prenatal testosterone exposure together with sex-typical behavioural tendencies^55,56^. Feminine evolutionary strategies are characterized by increased sharing, an earlier onset of theory of mind, more empathy and more donations^64^. Thus, it is expected that a lower prenatal testosterone exposure (higher 2D:4D) is associated with relatively more 1/1 choices. For the masculine strategy, it could be speculated that striving for exclusive ownership of a limited, valued good is beneficial in male-male competition and in terms of female choice. Therefore, lower 2D:4D ratios would be related with more 1/0 decisions. Both the outlined feminine and masculine strategies are reflected in a positive correlation between 2D:4D and the amount of 1/1 choices. A second, competing, line of argument is derived from the findings that higher prenatal testosterone exposure (lower 2D:4D) has been related to a stronger preference for fair contributions in adults^62^. In this regard, the 1/1 choice (as compared to the 1/0 alternative) represents the fair choice. If fairness rather than prosocial tendencies drive the decision process, one would expect a higher amount of 1/1 choices in children with lower digit ratios, i.e. a negative correlation between 2D:4D and the amount of 1/1 choices.

## Results

**Figure 2:**
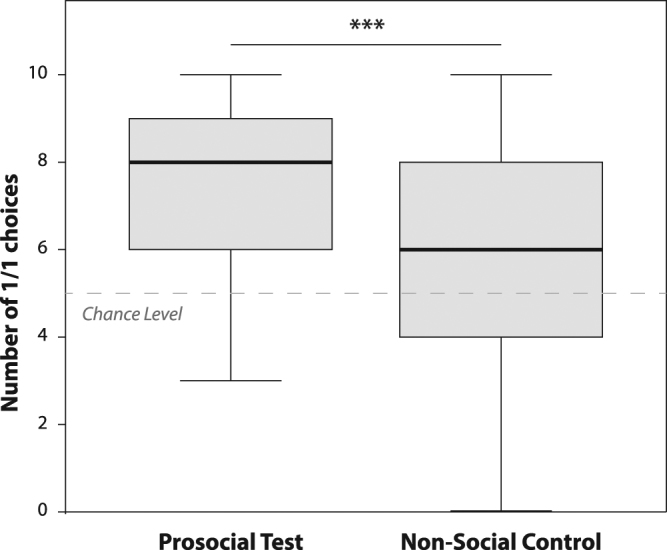
Boxplot of the number of 1/1 choices in the prosocial test and the non-social control (the bold line represents the median, the boxes represent 1^st^ and 3^rd^ quartile, the whiskers represent the minimum and maximum values). The dashed line indicates chance level. ***p ≤ 0.001.

The donor children chose the 1/1 option significantly above chance in both the test (total number of trials = 10; median_1/1_ = 8; one-sample Wilcoxon: N = 45, W = 863.5, p ≤ 0.001) and the non-social control (total number of trials = 10; median_1/1_ = 6; W = 537.0, p = 0.015), but they chose the 1/1 option significantly more often in the test than in the control condition (related-samples Wilcoxon: N = 45, 11 ties, T^+^ = 497.5, T^−^ = 97.5, p = 0.001; Fig. 2). Verbal and non-verbal (i.e. gesturing to the side with the 1/1 option) requests by the recipient occurred in only 5 instances throughout the study and are therefore not likely to have contributed to these differences. The children showed no side bias in either the test (median_right_ = 5; one-sample Wilcoxon: N = 45, W = 152.0, p = 0.767) or the non-social control (median_right_ = 5; W = 172.0, p = 0.307).

To control for the children’s general preference for choosing the 1/1 option, we calculated their prosocial tendencies by subtracting the number of 1/1 choices in the non-social control condition from the number of 1/1 choices in the test^13^. A positive prosocial tendency shows prosocial behaviour, whereas a negative prosocial tendency shows asocial behaviour. There was no significant difference in prosocial tendencies between children that were paired with friends (N = 23, median_1/1_ = 2) compared to those paired with non-friends (N = 22, median_1/1_ = 1; Mann-Whitney: U = 309.0, p = 0.198).

The donors’ prosocial tendencies were not correlated with their average number of interaction partners during the observation (Spearman’s rank correlation: N = 44, ρ = −0.003, p = 0.984). However, post-hoc analyses revealed that there was a negative correlation between the number of the donor’s interaction partners and the number of 1/1 choices in the test (ρ = −0.428, p = 0.004; p_Holm-Bonferroni_ = 0.012), as well as a non-significant trend of a negative correlation with the number of 1/1 choices in the control (ρ = −0.310, p = 0.040, p_Holm-Bonferroni_ = 0.080). There was no correlation between the participant’s prosocial tendencies and the number of children attending to him or her during the observation (N = 44, ρ = −0.123, p = 0.425) and the teachers’ social dominance ratings (N = 45, ρ = −0.114, p = 0.455). Teachers’ dominance ratings and the number of children attending to the participant were only correlated in males (N = 19, ρ = 0.616, p = 0.005), but not in females (N = 25, ρ = 0.088, p = 0.676).

**Figure 3:**
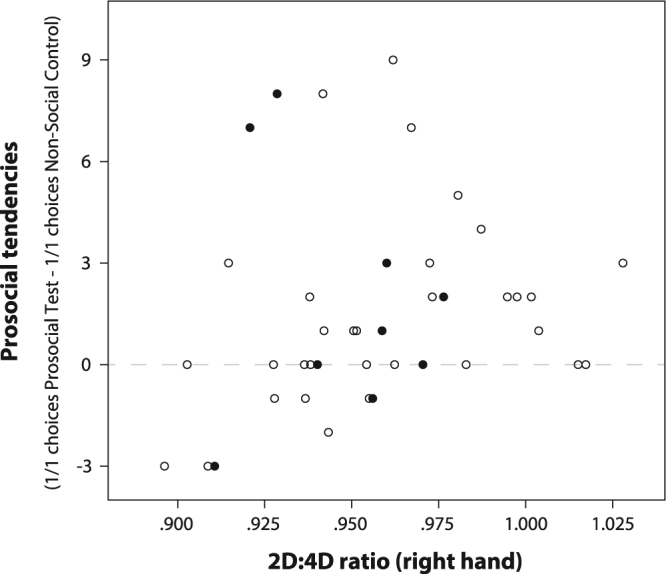
Scatter plot of the prosocial tendencies and right hand 2D:4D ratios. Black circles represent left-handed individuals (N = 9). The dashed line indicates an equal number of 1/1 choices in prosocial test and non-social control. There is a positive, linear correlation in the total sample (Spearman’s rank correlation: N = 41, ρ = 0.337, p = 0.031) and when excluding left-handed children (i.e., only white circles; N = 32, ρ = 0.419, p = 0.017).

There was a positive, linear correlation between the children’s prosocial tendencies and their right hand 2D:4D ratios, both when looking at the total sample (N = 41, ρ = 0.337, p = 0.031) and when excluding left-handed children (N = 32, ρ = 0.419, p = 0.017; Fig. 3). We found no sex difference in 2D:4D ratios (females: N = 24, mean = 0.959, sd = 0.030; males: N = 17, mean = 0.954, sd = 0.036; t-test: t = −0.544, p = 0.590). The 2D:4D ratios were not correlated with teachers’ dominance ratings (N = 41, ρ = −0.007, p = 0.963), the number of children attending to the participant (N = 40, ρ = 0.069, p = 0.672), or the average number of interaction partners during the observation (N = 40, ρ = 0.016, p = 0.921).

Age, sex, sequence of conditions, and number of trials in the warm-up phase had no effect on whether the children showed a prosocial response or not (Generalized linear model: N = 43, Wald Chi-Square; age: X^2^ = 0.473, p = 0.492; sex: X^2^ = 0.135, p = 0.714; sequence: X^2^ = 0.107, p = 0.744; warm-up: X^2^ = 0.569, p = 0.451). Descriptive statistics of all variables split by sex are shown in Table 1.

## Discussion

Our results show that 6–9-year-old children act prosocially when they are tested with familiar peers as social partners in a resource allocation paradigm that is not constrained by experimenter instructions, but by the physical properties of the apparatus. The children chose the prosocial option significantly more often when a recipient was present than when they were tested without a partner in the non-social control. Whether children were paired with a friend or a partner that they did not consider as their friend had little effect on their choices. However, their social status in the classroom seemed to influence their behaviour, albeit irrespective of the condition; i.e., post-hoc analyses revealed that in both the test and the non-social control, children with fewer social interaction partners chose the 1/1 option more often, although this trend was not significant in the non-social control. Further, the prosocial tendencies of the children in our test were negatively correlated with approximated prenatal androgen exposure; i.e., children with high 2D:4D ratios (indicating lower exposure to prenatal androgens) acted more prosocially than children with low 2D:4D ratios (indicating higher exposure to prenatal androgens).

Our results are in line with previous studies that employed more artificial resource allocation paradigms, where children were tested with recipients only represented by pictures at the time of testing^11,12^. However, in contrast to younger children, where prosocial behaviour is elicited by recipient requests^10^, verbal or non-verbal requests by the familiar recipient were extremely rare in our study and are therefore not likely to have contributed to the children’s prosocial choices. In a study that – similar to our own – used peer interaction partners and an apparatus whose physical properties dictated the resource allocation paradigm the researchers found that 7-year-old children only acted prosocially (i.e. choose to deliver the high-quality reward to the recipient) when they themselves also received the high-quality reward, but not when they received a low-quality reward^22^. This might have been due to the fact that children at this age are particularly motivated to equalize outcomes between interaction partners^12^. However, since in our study both the donor and the recipient always received the same reward item, it is not clear whether the children’s prosocial donations in our study were mainly motivated by a preference for equitable outcomes or not. In the same study, the children’s behaviour was comparable to that of adult chimpanzees, who also shared with their interaction partners as long as both of them received the high-quality reward, while human adults acted prosocially, irrespective of the quality of their own reward^22^. We further found that the children’s age had no effect on whether they acted prosocially or not, albeit the age range in our sample was rather small (6–9 years). These findings parallel the ones of House and colleagues^11^, who found no age effects in a sample with a much larger age range. Also, there was no difference between females and males in their propensity to share during the experiment.

Interestingly, the children in our study chose the 1/1 option above chance not only when they were paired with a recipient but also when they were tested alone. It is possible that children were influenced by the warm-up phase, which was used to allow the children to learn how the apparatus functions and to pay attention to the resource distribution on both sides. In this phase the barrier between the donor side and the recipient side was open and the children could obtain the rewards from both sides (see methods section for details). This phase was necessary to assess whether the children understood the resource allocation game in the absence of detailed experimenter instructions. Because the children could maximize their own payoff by choosing the 1/1 option in the warm-up phase, it is possible that they later retained a preference for this distribution. However – since we found no effect of the testing sequence (i.e. between the condition that directly followed the warm-up phase compared to the later condition) on children’s choices, it seems unlikely that there was a short-term carry-over effect from the warm-up phase, which selectively affected the first condition that the children were tested in. Additionally, in our paradigm there were no costs of choosing the 1/1 option in the non-social control. Nevertheless, this made it necessary to calculate the children’s prosocial tendency score to control for their general preference for choosing the 1/1 option.

Contrary to our predictions, we found that friendship had no effect on the children’s prosocial behaviour. In contrast to Moore^32^ the children that were paired with a friend as the recipient did not donate more reward items than those children paired with a non-friend. Importantly however, Moore did not only ask children to name their best friends, but also to indicate peers with whom they did not like to play. He found more prosocial donations to friends than to disliked peers, but donations to unfamiliar recipients were intermediate between friends and non-friends^32^. Therefore, it is possible that the effect was not only driven by stronger prosocial tendencies towards friends, but also by weaker prosocial tendencies towards disliked peers. We did not pair children in the non-friend group with disliked recipients, but simply with peers that they had not indicated as friends. This might be the reason why we did not detect any strong differences between the two groups. Also, other studies testing primary school children in direct interactions did not find that children benefitted their friends more^33–35^. School children are introduced to rules about social interactions (e.g. sharing equally, taking turns) and are usually encouraged to interact in a socio-positive way with both friends and non-friends. This might have also masked differences between the two groups in the school setting. Finally, since testing took place as a real-life interaction with a familiar peer, it is possible that an expectation of reciprocation affected the donor’s behaviour. Although none of the children that acted as donors had participated as recipient before, thereby excluding reciprocation during the experiment, the donors might have expected the recipients to reciprocate their prosocial acts during later in-class interactions. Children adjust their prosocial behaviour dependent on their interaction partners’ previous behaviour from 5 years of age^65^, and reciprocate prosocial acts^66^. In sum, it is not clear whether the children’s prosocial behaviour was motivated by a concern for the recipient or by an expectation for future reciprocation, and whether friendship plays a role in that. Interestingly, in long-tailed macaques prosocial choices are also not necessarily affected by close social relationships, but rather by the social status of the donor and/or the recipient^13,14^.

Similarly, in our study children’s social status in the class – as measured by their number of interaction partners^38^ – affected their choices in the resource allocation: the children with fewer interaction partners during the group observation, chose the 1/1 option more often than children who had more interaction partners, both during the test and – as a non-significant trend – during the non-social control. This indicates that children that were less popular as interaction partners had a general preference for the 1/1 option. In part these results are in line with a previous study^42^ that showed that children with a low social group status donated more rewards to an anonymous recipient than children with a high social status. However, since that study did not include a non-social control, it is not clear whether the donation of reward items was triggered by the recipient. Our results are, however, contrary to those found in long-tailed macaques, where in fact high ranking individuals were prosocial and low ranking were not, and where these dominant individuals seem to use prosocial acts to consolidate their rank^13^.

We found no correlation between the participants’ prosocial tendencies and their attention received from other individuals, which was also scored during the live observations. However, since visual attention is less conspicuous than direct social interaction, it is possible that we might have underestimated the measures of attention during the live observation. Therefore, in the future we would recommend video recordings of free play interactions, if consent can be obtained for such recordings. Finally, we found no correlation between teachers’ dominance ratings and the participants’ prosocial tendencies. However, teachers’ dominance ratings were only correlated with social status scores from the live observation in males but not in females. Therefore, it is possible that teachers’ ratings were not an accurate representation of social status across the whole sample. Taken together, our results show that the connection between social status and prosocial tendencies in children needs to be further investigated in the future.

We found that children with a low 2D:4D ratio (reflecting a high exposure to androgens *in utero*) were significantly less likely to donate stickers to their partners in the resource allocation game than those with higher 2D:4D (lower prenatal testosterone exposure) who chose the prosocial 1/1 option more. This favours the assumption of a moderately mediating role of prenatal testosterone exposure in social decision-making and supports the first set of predictions derived from an overall masculinisation with increasing prenatal testosterone exposure on multiple phenotypic levels (from appearance (e.g., facial shape^67^) to gender-typed play behaviour^68^). We actually find tentative support for both parts of the hypothesis: first, the amount of 1/1 choices is positively correlated with 2D:4D over a wide range of 2D:4D values. And second, following the pattern of monopolization and a competitive orientation, the three children who chose 1/0 clearly more often in the social than in the control condition (–3 values in Fig. 2, two males, one left-handed female) were among the four children with the lowest 2D:4D values. A study that looked at sex differences in play found that boys are generally more competitive and girls more cooperative in their play^68^. Although this study did not look at variation within the sexes, it can be taken as evidence that competition is the more masculine strategy – a tendency that might hold for a masculinising effect of higher prenatal testosterone exposure within each sex, too. Our results are in line with previous findings showing that children with lower 2D:4D rations are rated as less prosocial by their teachers and caregivers^61,69^. While adults with low 2D:4D ratios prefer to make fair contributions in an anonymous public goods game^62^, this pattern is reversed after exposure to aggression cues: in this context participants with low 2D:4D ratios acted less and participants with high 2D:4D ratios acted more prosocially^63^. Those aggression cues might have triggered more competitive behaviour in the participants with low 2D:4D ratios. Similarly, it is possible that our set-up with a familiar peer as a direct interaction partner was perceived as a particularly competitive situation. Therefore, it would be important to also investigate the association between prenatal androgen exposure and children’s prosocial resource allocation in an anonymous situation.

Although it has been argued that prenatal androgen exposure is positively related to social status in humans (especially with regard to sports performance and financial markets, summarized in^70^) and rhesus macaques^71^, we found no correlation between the children’s 2D:4D ratios and their scores for social dominance and within-class status (i.e., teachers’ dominance ratings, the number of children attending to the participant, the average number of interaction partners during the observation). However, in humans, the relationship between low 2D:4D and self-rated dominance in males only emerged when the co-player was given a dominant face^70^. Thus a dominant behavioural pattern might blur when an opinion is formed based on interactions with many different interaction partners as is the case for the context in which the children’s teachers had to assess their dominance. Furthermore, we also did not find a significant sex difference in 2D:4D. This is not too surprising, given that between-sex overlap and within-sex variability are usually large^54^. Also, in a longitudinal study using radiographs, the sex difference in 2D:4D was not significant until the age of 9 years^51^, which is slightly older than our sample.

In sum, we showed that 6–9-year-old children behaved prosocially in a direct peer-to-peer interaction in a resource allocation paradigm dictated by the apparatus’ physical properties, and that their prosocial behaviour was negatively influenced by their prenatal testosterone exposure (assessed via 2D:4D) and by their social status in the class – although the latter effect was only found in the prosocial test condition. However, since in our *prosocial game* there was no cost for choosing the 1/1 option – neither in the prosocial test, nor in the non-social control – future research should investigate children’s choices using the same paradigm for a *costly sharing game* (i.e., the donors are required to choose between the 1/1 option and a 2/0 option, which would deliver two reward items to them) and an *envy game* (i.e., the donors are required to choose between the 1/1 option and a 1/2 option, which would deliver only one reward item to them, but two to the recipient). It is possible that under those circumstances, the children would be more selective in whom they would benefit, which would help scrutinizing the children’s social strategies as a function of friendship and social status. Since both the costly sharing and the envy situation will be likely perceived as more competitive by the participants, it might also amplify the effects of social status and prenatal testosterone exposure. Further, it might be interesting to broaden the age range: testing younger children could help to clarify at which age prosocial tendencies emerge. Testing adults in the same paradigm on the other hand might clarify whether similar physiological and status effects exist at a post-pubertal age. Finally, also in non-human primates and rodents 2D:4D ratios seem to reflect prenatal testosterone exposure^47–50,71^ and have been found to be related to sexual dimorphism^72^ and social dominance^71^. Therefore, a comparative approach might highlight the phylogenetic origins of prenatal androgens as physiological modulators in the relationship between social status and prosociality.

## Methods

### Participants

Participants were recruited from four first and second grade classes from two primary schools in Vienna. The children came from mixed socio-economic backgrounds. The sample consisted of 48 children (27 females and 21 males; age: mean ± sd = 8 years ± 9 months, min = 6.7 years, max = 9.8 years). Three children failed the warm-up in the resource allocation experiment and were excluded from data analysis (see detailed information below). Of the 45 remaining children, seven children were missing data from one other part of the study (2D:4D, N = 4; age, N = 2; observations, N = 1; see also Table 1). To maximise sample size, these children were included in those analyses, where we had obtained data. Analysis of only the children from whom we had obtained a full data set (N = 38) showed fully equivalent results (see Supplementary Results file).

The ethics committee of the University of Vienna and the Vienna School Board had approved our study (Ref. No. 00103). Informed consent forms had been obtained from all participants’ legal guardians and all procedures were carried out in accordance with the approved guidelines and the Helsinki agreement.

### Resource Allocation Experiment

The apparatus for the resource allocation experiment consisted of two wooden boxes (84 cm × 20 cm each) fixed on a wooden plate (100 × 80 cm, Fig. 1b). The two boxes were closed seamlessly on top with transparent Plexiglas lids, so that children could look inside the boxes, but not open them. On the donor’s side were two coin receptacles, one in front of each box. When inserting a coin into the coin receptacle, the Plexi lid of the corresponding box lifted so that the children could get access to the contents inside the box. In the middle – separating the donor compartment from the recipient compartment – there was a non-transparent chamber containing the electronic devices, which opened the lid. The mechanism was operated by the experimenter by means of a hidden remote control. In each trial, the participant received one green plastic coin, which allowed him or her to open one of the two wooden boxes. The reward distribution was always the 1/1 option in one box (i.e., one reward item on the donor side and one reward item on the recipient’s side) and the 1/0 option in the other box (i.e., one reward item on the donor side, but no reward item on the recipient’s side). The experimenter used three identical stickers in each trial. The experimenter showed the two stickers of the 1/1 option and the one sticker of the 1/0 option to the child before placing them in the respective boxes, so that the reward distribution was obvious. He always started with placing the rewards in the right box. Which box contained which option was counterbalanced across trials. Testing took place outside the classroom in a quiet room. The apparatus was placed on a table. Additional tables could be used to build a barrier and prevent donor children from getting to the recipient’s side of the apparatus (Fig. 1a).

The donor first started with a warm-up phase, designed to make sure that the child understood the apparatus and also paid attention to the recipient compartments. In this phase the barrier between the two sides was open and the donor could retrieve stickers from both the donor and the recipient side of the chosen box. The child was told that the boxes could be opened with the coin and that he or she was allowed to explore the apparatus freely. In this phase, the participant could maximize his or her payoff by choosing the 1/1 option, because both stickers could be retrieved. If the participant chose the 1/1 option in every trial of the first 4 trials, he or she proceeded to the test phase (N = 36). If the participant did not choose the 1/1 option in every trial, he or she received another block of 4 trials and proceeded to the test phase when the 1/1 option was chosen in all 4 trials (N = 9). Only three children failed the criterion in both blocks of warm-up trials and did not participate in the test phase.

In the test phase the experimenter closed the barrier between the donor side and the recipient side, so that the donor was physically prevented to retrieve reward items from the recipient’s side. Each participant was tested in two conditions: in the “prosocial test” a recipient child was present on the recipient’s side and could retrieve a sticker, if the donor chose the 1/1 option. In the “non-social control” there was no recipient child present, but the donor could not obtain the sticker on the recipient’s side (i.e., the sticker was returned to the experimenter’s stash). The sequence of the two conditions was counterbalanced across children. In each condition, the donor received 10 consecutive trials. Some children played the roles of both donor and recipient in separate sessions. However – to avoid individual^73^ or generalized^74^ reciprocity effects – the children always played the role of donor first and never acted as a donor after having participated as a recipient.

### Observational Procedures

Children were observed live during free play periods (i.e. during recess), because we could not obtain consent for video recordings from all participants’ legal guardians. We used instantaneous sampling at sampling intervals of 10 seconds. One focal child was observed at each sampling point and the sequence of children was determined randomly and fixed within each class. At each sampling point, we recorded two variables for the focal child: the number of interaction partners (i.e., other children at arm’s length) and the number of children attending to the focal child (cf.^38^). In total, each child was observed four times, twice on each of two separate days. We calculated the average number of interaction partners and number of children attending to the participant per sampling point.

### 2D:4D ratio measurements

We scanned the palmar surface of both hands of every child placed in a flat position without pressure on a flatbed scanner (Canon LIDE 110) with an attached ruler. One rater measured the second and fourth digits of both hands of each child from the ventral-most proximal crease to the tip of the finger with the program “TpsDig2”^75^. Each digit was measured twice, and we computed the mean of both measurements. If the first and the second measurement were more than 0.5 mm apart, the rater performed a third measurement and the two closer scores were retained (N = 3). We computed the 2D:4D ratio by dividing the length of the second by the length of the fourth digit. The scans of two participants were of poor quality with resulting digit ratios more than two standard deviations below and above the sample mean, respectively. Therefore, these two scans were discarded from the sample.

Intra- and inter-observer reliability were calculated for the right and left hands of the first 32 individuals. The two measurements of the first rater were highly correlated (Spearman; N = 128, r = 0.997, p < 0.001) and there was no significant difference between first and second measurements (Pillai trace = 0.005, F = 1.221, df = (1, 254), p = 0.270). Additionally, the measurements were highly correlated with those taken by a second, independent rater (Spearman; N = 128, r = 0.979, p < 0.001). There was no significant difference between the two raters (Pillai trace = 0.006, F = 1.514, df = (1, 254), p = 0.220) and 98% of the measurements had a difference of ≤0.5 mm.

Since the 2D:4D ratio of the right hand has been argued to show a stronger association with prenatal androgens than those of the left hand^53^ we used only the measures from each child’s right hand for the analysis.

### Questionnaires

The teacher questionnaire was created with SoSci Survey^76^ and made available to the teachers on www.soscisurvey.com. The classroom teachers rated each child’s perceived social dominance on a continuous, graphic rating scale ranging from “strongly disagree” to “strongly agree” for four items (i.e., “assertive”, “dominates classmates,” “tells others what to do,” “stands up for self” cf.^77^; Cronbach’s α = 0.93). Moreover, classroom teachers were asked to identify up to five best friends for each child.

Additionally, children were asked to identify their best friends in the classroom in a paper questionnaire. There was a significant agreement between friend ratings provided by the children and by the teachers (Pearson’s chi-squared test: N = 47, X^2^ = 6.22, p = 0.013). Therefore, we decided to use only children’s ratings for further analyses.

### Data analysis

Our dependent variables from the resource allocation experiment were the “number of 1/1 choices” in the test and the non-social control (each ranging from 0 to 10). To control for a general preference for the 1/1 option we calculated the “prosocial tendencies” by subtracting the number of 1/1 choices in the non-social control from the number of 1/1 choices in the test (cf.^13^).

Additionally, we scored whether children showed a prosocial response (i.e. when they chose the 1/1 option more often in the test than in the non-social control; score = 1) or not (i.e. when they chose the 1/1 option equally or less often in the test than in the non-social control; score = 0). With this binary response variable we calculated a Generalized Linear Model with age, sex, sequence of conditions, and number of warm-up trials as predictors.

In each trial, we scored whether the recipient requested the 1/1 option verbally (e.g. asking the donor verbally to open one specific box, giving statements about the stickers in the boxes without making precise requests) or non-verbally (e.g. gesturing towards a specific box).

Of all the variables, only 2D:4D ratio and age were normally distributed. Therefore, we used non-parametric statistics in all analyses, apart from testing for a sex difference in 2D:4D ratio. All tests were two-tailed and we set alpha to 0.05. We used Holm-Bonferroni^78^ correction for calculating corresponding p-values when multiple comparisons were made on one set of data.

### Data availability

The dataset generated during the study is available from the corresponding author on reasonable request.

## Electronic supplementary material

Supplementary Results
