# Supplementary material for: Social status and prenatal testosterone exposure assessed via second-to-fourth digit ratio affect 6–9-year-old children’s prosocial choices
Source: Sci Rep. 2018 Jun 15;8:9198. doi: 10.1038/s41598-018-27468-0 (PMC6004003; doi:10.1038/s41598-018-27468-0)
Supplement: Supplementary file 1 — Supplementary Results [file 41598_2018_27468_MOESM1_ESM.pdf]

Social status and prenatal testosterone exposure assessed via second-to-fourth digit ratio affect 6–9-year-old children's prosocial choices

Lisa Horn, Niklas A. Hungerländer, Sonja Windhager, Thomas Bugnyar, Jorg J.M. Massen

Supplementary Results

Here, we present all analyses of the main article, conducted on the sub-sample of children from whom we had obtained a full data set (N=38; 22 females and 16 males).

Number of 1/1 choices in the test and the non-social control

The donor children chose the 1/1 option significantly above chance in both the test (total number of trials=10; $median_{1/1}$=8; one-sample Wilcoxon: W=597.0, p≤0.001) and the non-social control (total number of trials=10; $median_{1/1}$=6; W=373.5, p=0.039), but they chose the 1/1 option significantly more often in the test than in the control condition (related-samples Wilcoxon: 10 ties, T+=332, T-=74, p=0.003). The children showed no side bias in either the test ($median_{right}$=5; one-sample Wilcoxon: W=122.5, p=0.891) or the non-social control ($median_{right}$=5; W=110.0, p=0.377).

Prosocial tendencies and friendship, interaction partners, other children attending, and dominance ratings

There was no significant difference in prosocial tendencies between children that were paired with friends (N=20, $median_{1/1}$=1.5) compared to those paired with non-friends (N=18, $median_{1/1}$=1; Mann-Whitney: U=204.5, p=0.478).

The donors' prosocial tendencies were not correlated with their average number of interaction partners during the observation (Spearman's rank correlation: ρ=-0.067, p=0.691). However, post-hoc analysis revealed that there was a negative correlation between the number of the donor's interaction partners and the number of 1/1 choices in the test (ρ=-0.546, p≤0.001; $p_{Holm\text{-}Bonferroni}$≤0.001), as well as a non-significant trend of a negative correlation with the number of 1/1 choices in the control (ρ=-0.326, p=0.046, $p_{Holm\text{-}Bonferroni}$=0.092).

There was no correlation between the participant's prosocial tendencies and the number of children attending to him or her during the observation (ρ=-0.179, p=0.282) and the teachers' social dominance ratings (ρ=-0.100, p=0.459). Teachers' dominance ratings and the number of children attending to the participant were only correlated in males (N=16, ρ=0.712, p=0.002), but not in females (N=22, ρ=-0.043, p=0.850).

Prosocial tendencies and 2D:4D

There was a positive, linear correlation between the children's prosocial tendencies and their right hand 2D:4D ratios, both when looking at the total sample (N=38, ρ=0.364, p=0.025) and when excluding left-handed children (N=29, ρ=0.466, p=0.011). We found no sex difference in 2D:4D ratios (females: N=22, mean=0.956, sd=0.029; males: N=16, mean=0.954, sd=0.037; t-test: t=-0.162, p=0.873). The 2D:4D ratios were not correlated with teachers' dominance ratings (ρ=0.047, p=0.779), the number of children attending to the participant (ρ=0.039, p=0.816), or the average number of interaction partners during the observation (ρ=-0.006, p=0.971).

Prosocial response and age, sex, sequence of conditions and number of trials in the warm-up

Age, sex, sequence of conditions, and number of trials in the warm-up phase had no effect on whether the children showed a prosocial response or not (Generalized linear model: Wald Chi-Square; age: X^2=0.751, p=0.386; sex: X^2=0.000, p=0.997; sequence: X^2=0.126, p=0.723; warm-up: X^2=0.122, p=0.726).
